# Severe velopharyngeal stenosis following repeated adenotonsillectomy in a child: a rare diagnostic and surgical challenge

**DOI:** 10.1093/jscr/rjaf621

**Published:** 2025-08-13

**Authors:** Hind El Yousfi, Nawfal Fejjal, Ali El Ayoubi

**Affiliations:** Laboratory of Anatomy and Experimental Surgery, Faculty of Medicine and Pharmacy, Mohammed V University, Avenue Mohamed Belarbi Alaoui, Al Irfane, Rabat 10000, Morocco; Plastic Surgery Unit, Surgical Department C, Children’s Hospital, CHU Ibn Sina, BP 6527, Lemfadel Cherkaoui Street, Souissi District, Rabat, Morocco; Faculty of Medicine and Pharmacy, Mohammed V University, Avenue des Nations Unies, Agdal, Rabat 10000, Morocco; Plastic Surgery Unit, Surgical Department C, Children’s Hospital, CHU Ibn Sina, BP 6527, Lemfadel Cherkaoui Street, Souissi District, Rabat, Morocco; Faculty of Medicine and Pharmacy, Mohammed V University, Avenue des Nations Unies, Agdal, Rabat 10000, Morocco; Laboratory of Anatomy and Experimental Surgery, Faculty of Medicine and Pharmacy, Mohammed V University, Avenue Mohamed Belarbi Alaoui, Al Irfane, Rabat 10000, Morocco; Faculty of Medicine and Pharmacy, Mohammed V University, Avenue des Nations Unies, Agdal, Rabat 10000, Morocco; Department of Pediatric Surgery, Children’s Hospital, CHU Ibn Sina, BP 6527, Lemfadel Cherkaoui Street, Souissi District, Rabat 10000, Morocco

**Keywords:** velopharyngeal stenosis, repeated tonsillectomy, postoperative complication, child, pediatric otolaryngology

## Abstract

Severe complications following tonsillectomy are rare in the pediatric population. However, repeated oropharyngeal surgeries can occasionally result in velopharyngeal stenosis. We report the case of a 7-year-old patient from Côte d'Ivoire who underwent two tonsillectomies 5 months apart, along with an adenoidectomy. Subsequently, she developed hypernasal speech, chronic nasal obstruction, dysphagia to solids, and mild respiratory discomfort. Nasal endoscopy revealed a postoperative velopharyngeal stenosis characterized by fibrous narrowing between the soft palate and the posterior pharyngeal wall. A corrective surgical procedure was performed, including dissection of fibrous adhesions and anatomical reconstruction of the soft palate. The postoperative course was favorable, with improved phonation, resolution of respiratory symptoms, and disappearance of dysphagia. This rare case highlights the importance of early endoscopic diagnosis and appropriate surgical management to prevent long-term functional sequelae.

## Introduction

Tonsillectomy and adenoidectomy are common ear, nose, and throat (ENT) procedures in children, generally well tolerated with few complications. Usual adverse effects include pain, bleeding, or infection. In contrast, severe structural complications like velopharyngeal stenosis are extremely rare. Velopharyngeal stenosis is an abnormal narrowing between the soft palate and posterior pharyngeal wall. It may cause hypernasality, nasal obstruction, or difficulty with speech and swallowing. It is more frequently reported in adults, especially after radiotherapy or complex pharyngeal surgery. Pediatric cases following tonsillectomy or adenoidectomy are exceptional.

We report a case of a 7-year-old girl who developed velopharyngeal stenosis after two closely spaced tonsillectomies and an adenoidectomy. This case highlights the need to consider this rare entity when voice or breathing disorders persist postoperatively. Pathophysiology, diagnosis, and treatment are discussed.

## Case report

A 7-year-old girl from Côte d'Ivoire, with no relevant history, underwent two tonsillectomies at age 5, 5 months apart, for recurrent tonsillitis and hypertrophy. An adenoidectomy was added during the second surgery (operative reports were unavailable).

Over the following months, she developed nasal obstruction, hypernasal speech, exertional dyspnea, dysphagia for solids, and pharyngeal fullness. Preoperatively, she had presented with severe hypernasality, with a velopharyngeal insufficiency (VPI) score of 3/3 on a standardized perceptual scale, and she had a functional oral intake scale (FOIS) level of 4 (oral intake limited to liquids/semi-liquids). She was referred to a pediatric otolaryngologist (ENT specialist).

Clinical examination revealed marked hypernasality. Endoscopy showed fibrous narrowing at the velopharyngeal port, with adhesion of the soft palate to the posterior pharyngeal wall. No infection or inflammation was noted.

Diagnosis of post-surgical velopharyngeal stenosis, likely due to fibrosis from repeated surgical trauma, was made. Surgery was indicated.

The procedure was performed under general anesthesia, with infiltration of the soft palate mucosa using lidocaine with epinephrine.

**Figure 1:**
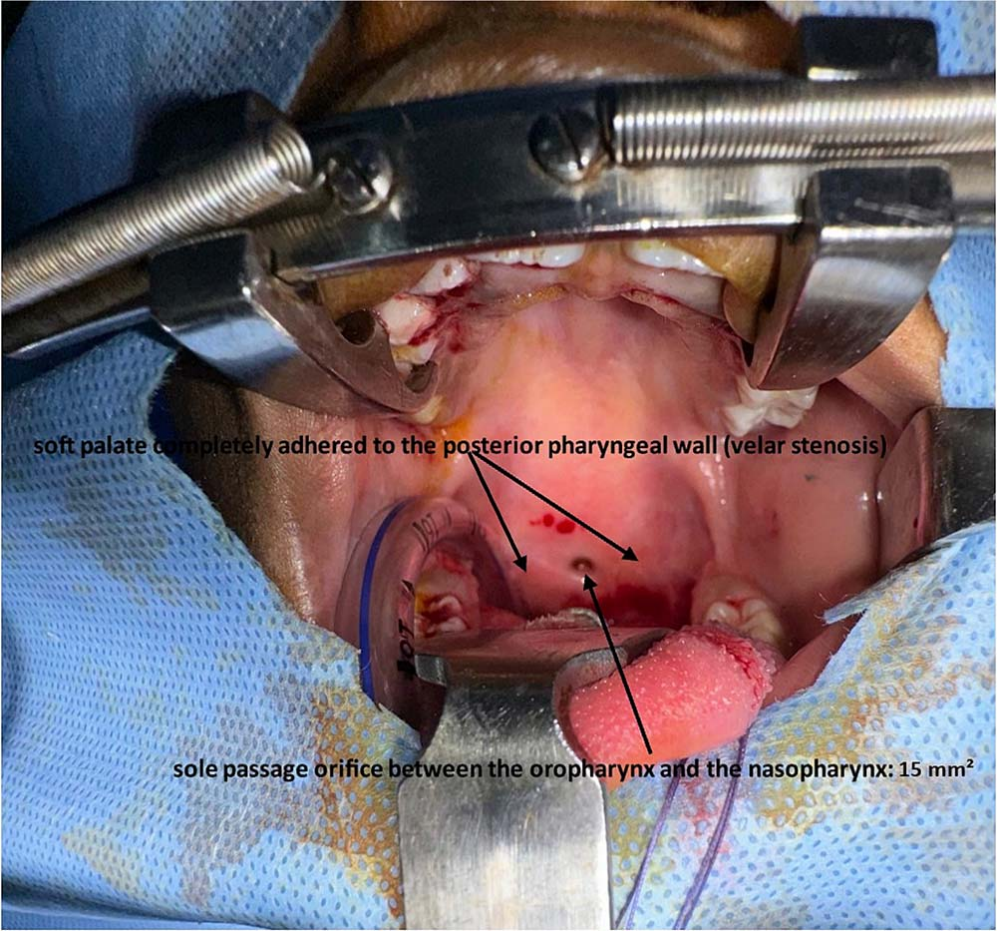
Intraoperative view showing severe velopharyngeal stenosis with complete adhesion between the soft palate and the posterior pharyngeal wall, leaving only a small central opening estimated at approximately 15 mm^2^ (arrow).

The initial exploration allowed for a precise visualization of the velar stenosis through both the oral route and nasal endoscopy. It revealed a complete adhesion of the soft palate to the posterior pharyngeal wall, with the presence of a single residual orifice establishing communication between the oropharynx and the nasopharynx. This orifice was estimated to measure approximately 15 mm^2^, indicating a severely narrowed velopharyngeal port (Fig. 1).

**Figure 2:**
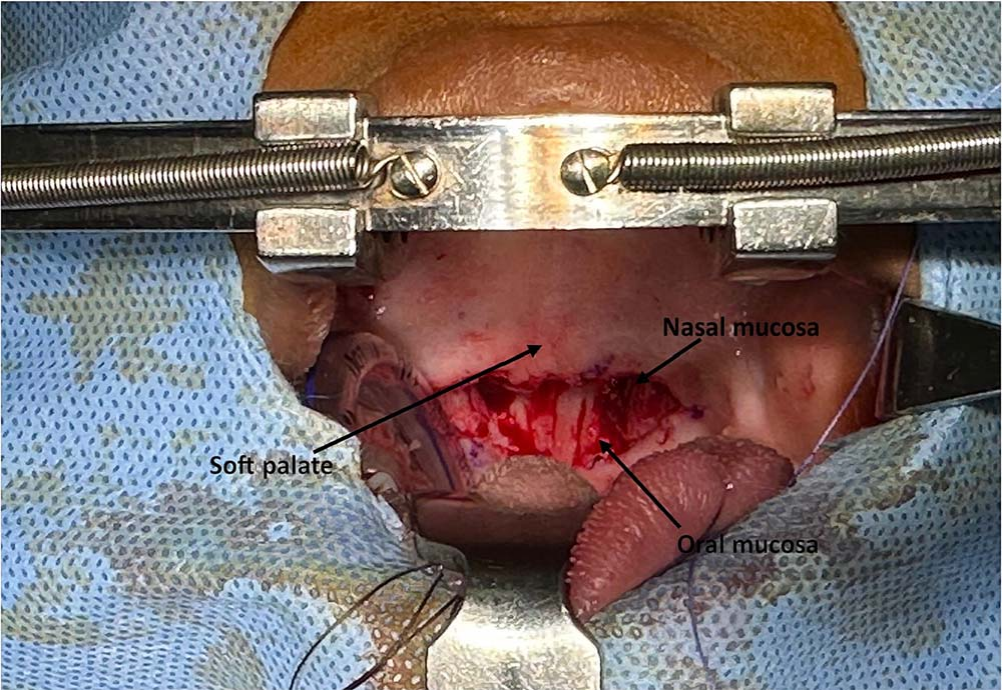
The intraoperative view following the release of the soft palate stenosis from the posterior wall of the oropharynx, illustrating the distinction between the nasal and oral mucosa of the soft palate.

A meticulous dissection of the fibrous planes was undertaken, which allowed for the release of the synechiae responsible for the velopharyngeal obstruction, and the distinct separation of the nasal and oral mucosa at the level of the soft palate. This procedure facilitated a clear identification of the two mucosal surfaces (Fig. 2).

**Figure 3:**
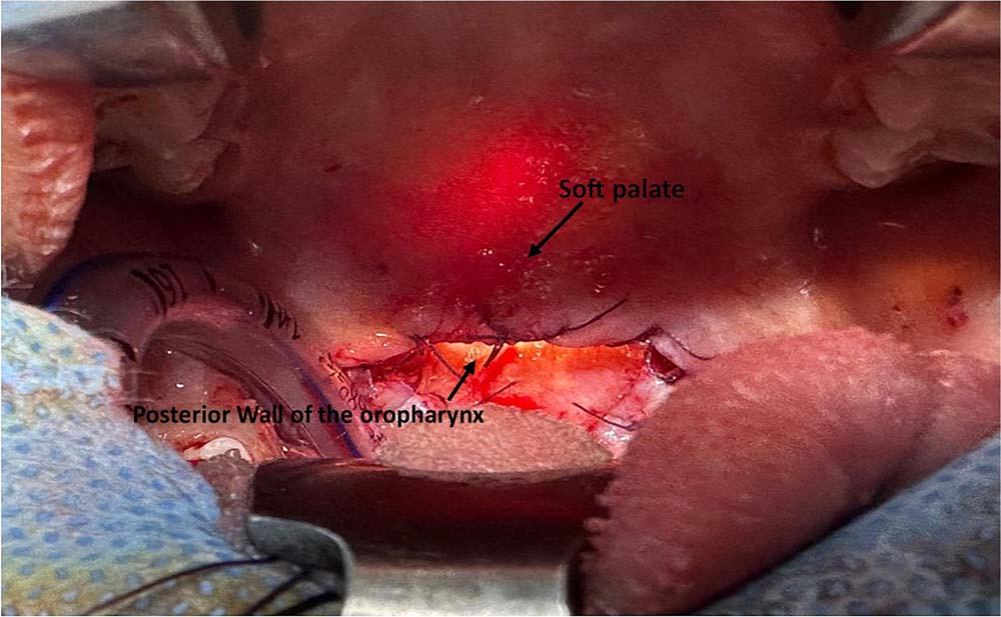
Intraoperative view showing the final result after reconstruction of the soft palate.

An anatomical reconstruction of the soft palate was then performed, involving layered closure of the muscular and mucosal planes to restore velopharyngeal patency and prevent recurrence (Fig. 3).

**Figure 4:**
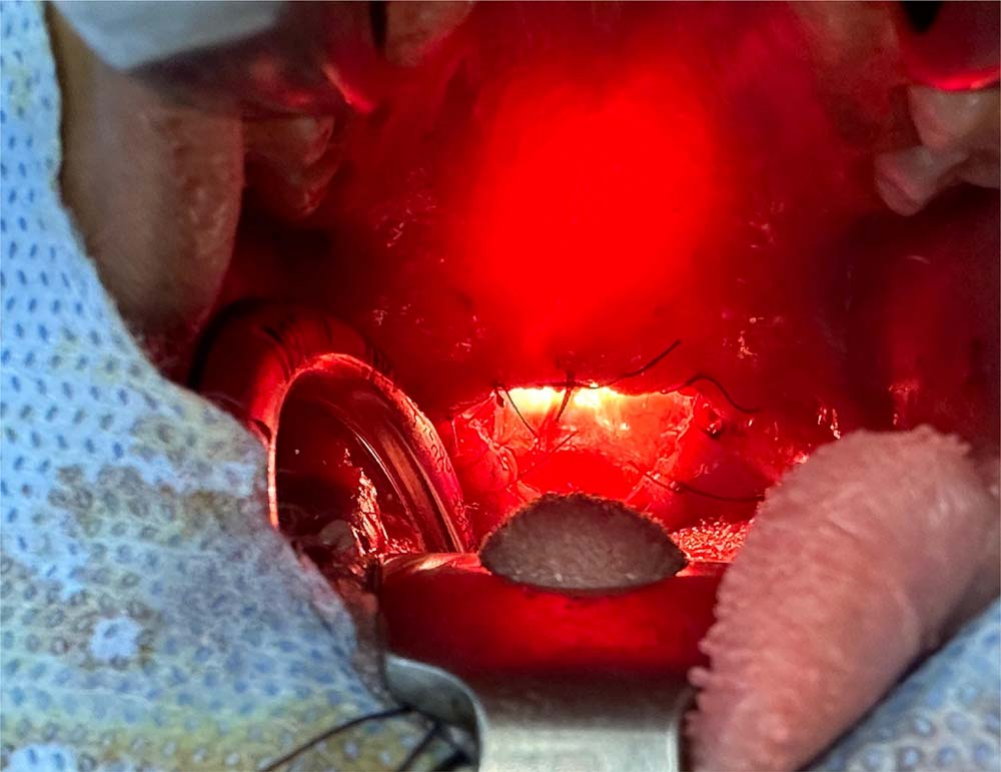
Intraoperative view confirming the patency of the upper aerodigestive tract via oral and nasal endoscopic approaches.

Intraoperative assessment of upper aerodigestive tract patency was conducted through both oral and endoscopic nasal approaches, confirming a satisfactory outcome (Fig. 4).

**Figure 5:**
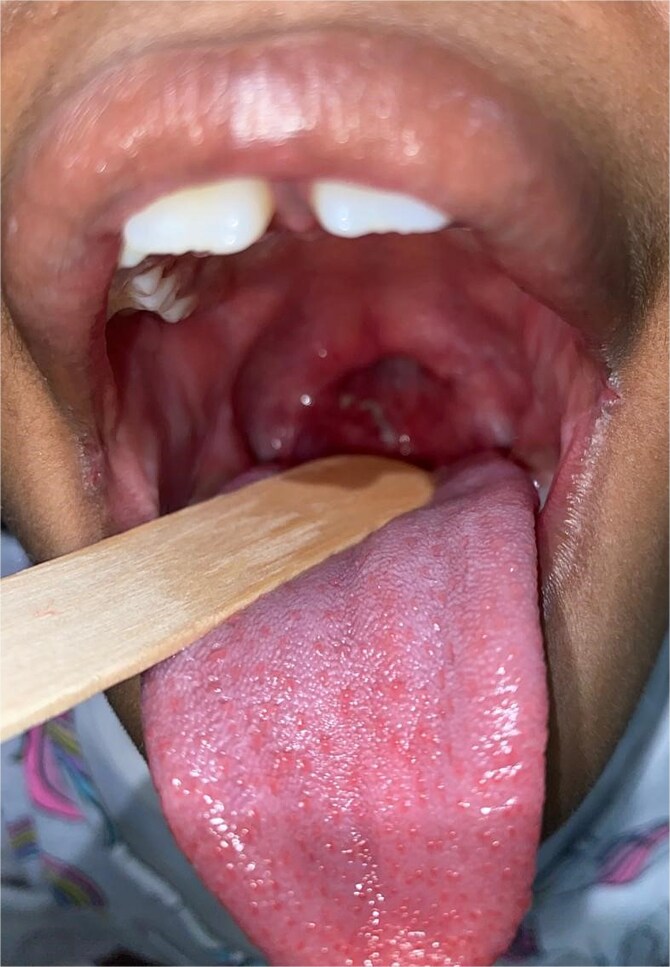
Two-month postoperative follow-up image showing favorable anatomical and functional outcome of the soft palate and oropharynx.

**Figure 6:**
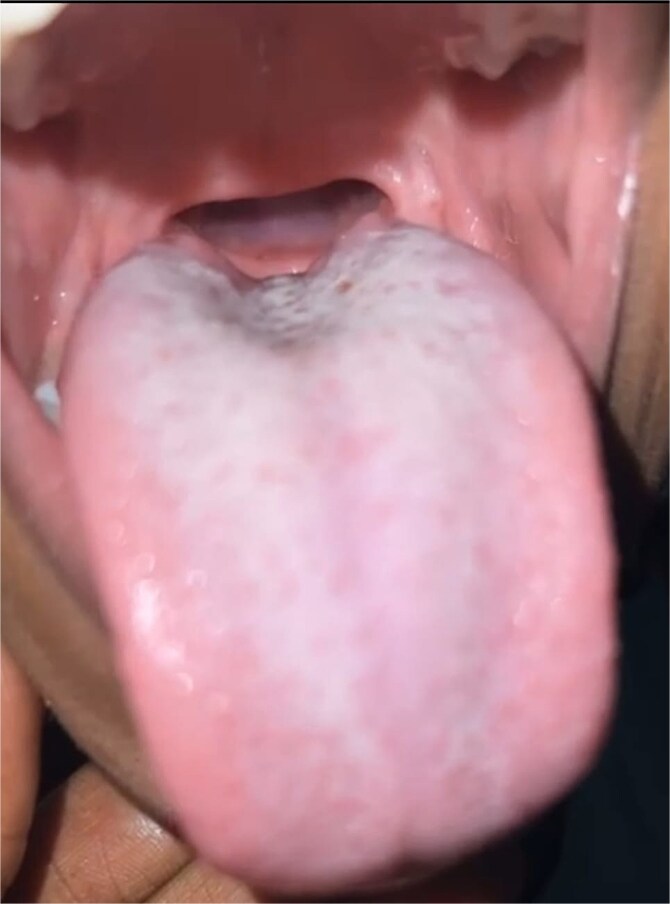
The follow-up image of the soft palate and oropharynx at 10 months post-intervention demonstrates satisfactory anatomical and functional progress.

Recovery was favorable. At 2 months, wound healing was good (Figs 5 and 6), speech improved, respiratory, and swallowing symptoms resolved. The VPI score decreased to 1/3, FOIS rose to 6.

## Discussion

Velopharyngeal stenosis is an extremely rare complication after tonsillectomy, especially in children. Most cases in the literature concern adults undergoing head and neck cancer surgery or post-radiation fibrosis, where scarring is an expected sequela [1]. In children, post-adenotonsillectomy stenosis remains exceptional, with few reported cases [2]. Its pathophysiology is not clearly defined but appears multifactorial. In our case, two tonsillectomies within 5 months, combined with adenoidectomy, likely amplified mucosal trauma and disrupted healing, predisposing to excessive fibrosis.

Though the exact etiology remains uncertain due to missing operative records, repeated trauma likely caused cumulative micro-injuries to oropharyngeal tissues. Intraoperative bleeding may have required cauterization, causing thermal injury. These burns could have induced tissue necrosis, followed by abnormal scarring and fibrous adhesions between the tonsillar pillars, soft palate, and posterior pharyngeal wall. Additionally, adenoidectomy may have caused nasopharyngeal trauma, fostering cicatricial bridges between adjacent structures—explaining the progressive stenosis.

Histologically, velopharyngeal stenosis involves dense fibrous bands tethering the soft palate, narrowing, or obliterating the velopharyngeal port [3]. Risk factors for exaggerated fibrosis include repetitive surgery, chronic inflammation, healing variability, and lack of mucosal separation [4].

Diagnosis is based on clinical findings—hypernasality, nasal obstruction, dysphagia—and endoscopy. Flexible nasoendoscopy offers direct visualization of the port and is essential for grading. In our case, nasal and oral endoscopy enabled complete evaluation and guided surgical planning.

Differential diagnosis includes congenital anomalies (e.g. submucosal cleft), craniofacial syndromes, neuromuscular disorders causing velopharyngeal insufficiency, obstructive lesions (e.g. adenoidal hypertrophy, benign tumors), and sequelae of chemical or thermal burns [5, 6].

Management is surgical, adapted to the extent and nature of stenosis. Options include intravelar veloplasty, pharyngeal flap surgery, and lateral or sphincter pharyngoplasty [7]. In our case, meticulous dissection of adhesions and layered soft palate reconstruction restored anatomical separation and velopharyngeal patency. Intraoperative endoscopic reassessment confirmed success.

Recurrence appears less frequent in children, likely due to better tissue regeneration and absence of comorbidities, such as radiotherapy or cancer surgery [8, 9]. Our patient remained asymptomatic after 10 months, with no restenosis.

Adjunctive therapies like mitomycin C or stenting were not used. The stenosis was localized, fibrous, and surgically correctable. Mitomycin C, although used in airway surgery, has controversial efficacy in pharyngeal settings and may impair healing [10, 11]. Stents were avoided due to poor pediatric tolerance, infection risk, and possible pressure necrosis [12].

This case underlines key considerations:

(1) Severe fibrosis may occur after routine ENT surgery.

(2) Persistent hypernasality or dysphagia warrants suspicion.

(3) Nasoendoscopy is crucial for diagnosis and planning.

(4) Individualized surgery based on anatomy can be successful.

The favorable outcome—improved speech, resolved respiratory symptoms, normalized swallowing—confirms the value of early diagnosis and appropriate surgical treatment. This report contributes to the limited pediatric literature on this rare but impactful complication.

## Conclusion

Post-adenotonsillectomy velopharyngeal stenosis is rare but significant. Early endoscopic diagnosis and surgical correction are essential. The rarity of such cases outside oncologic settings justifies publication. Favorable outcomes support prompt, tailored management.
